# A therapeutic ERCP in an only 5.9 kg infant with obstruction jaundice using JF-260V duodenoscope

**DOI:** 10.1186/s12887-024-04765-4

**Published:** 2024-04-27

**Authors:** Tian Zhang, Yijun Shu, Hao Weng, Mingzhe Weng, Ying Zhou, Wei Cai, Xuefeng Wang

**Affiliations:** 1 Department of Pediatric Surgery, Xinhua Hospital Affiliated to Shanghai Jiao Tong University School of Medicine, 1665 KongJiang Street, Shanghai, 200092 China; 2 Department of General Surgery, Xinhua Hospital Affiliated to Shanghai Jiao Tong University School of Medicine, 1665 Kongjiang Street, Shanghai, 200092 China

**Keywords:** ERCP, Infant, Biliary obstruction

## Abstract

**Background:**

Endoscopic retrograde cholangiopancreatography (ERCP) has found extensive use in pediatric patients; however, challenges persist in the application of therapeutic ERCP in infants.

**Case presentation:**

This case report details the presentation of a 5.9-kilogram infant with obstructive jaundice and suspected hemolytic anemia who underwent ERCP to alleviate biliary obstruction. The infant was admitted due to clay-colored stools, jaundice, and liver injury. Ultrasound and magnetic resonance cholangiopancreatography (MRCP) revealed dilation of the common bile duct (CBD) accompanied by the presence of stones. ERCP was conducted using a JF-260V duodenoscope under general anesthesia. Successful stone extraction and biliary drainage were achieved.

**Conclusions:**

In centers with considerable expertise in ERCP and pediatric anesthesia, the use of a conventional adult duodenoscope for therapeutic ERCP in infants can be considered safe and feasible, provided careful and stringent patient selection criteria are applied. In the future, clear guidelines and standardized protocols for the indications and procedures of pediatric ERCP should be established.

## Background

Endoscopic retrograde cholangiopancreatography (ERCP) has long been employed for diagnosing and treating biliary and pancreatic diseases over the course of several decades [1]. The utilization of ERCP in pediatric cases has experienced a significant upsurge in recent years [2, 3]. Our institution has conducted nearly 500 therapeutic pediatric ERCP procedures. The majority of endoscopic procedures and devices employed in ERCP are intended for adult patients. Consequently, ERCP poses a considerable challenge when performed on underweight children, particularly newborns and infants [4]. While there have been reports of successful ERCP procedures in neonates, the duodenoscope PJF-160 used in these cases cannot facilitate therapeutic ERCP due to its limited 2mm diameter forceps channel [5, 6]. Recently, our center successfully performed a therapeutic ERCP on an infant weighing only 5.9 kg. In this report, we present a case study and a discussion regarding the application of pediatric ERCP.

## Case presentation

A 5-month-old girl was admitted to our department due to a complaint of "stool color has been light for more than 20 days". During the 1.5 months prior to this hospitalization, the child sought medical attention at a local hospital due to poor weight gain. Abdominal ultrasound revealed gallbladder stones. She had no history of blood transfusion, no apparent history of medication, infection, or other relevant medical conditions. As the child had no apparent symptoms at that time, no specific treatment was initiated. About three weeks later, the child's parents noticed that the color of the stool had become lighter and brought the child back to the hospital for further evaluation. After hospital admission assessment, simultaneous presence of skin and scleral jaundice is noted. The girl had undergone an ultrasound examination, which revealed a mild 0.4cm dilation of the common bile duct (CBD) and suspected stones in both the CBD and gallbladder. The infant also presented with significant liver function abnormalities.

**Fig. 1:**
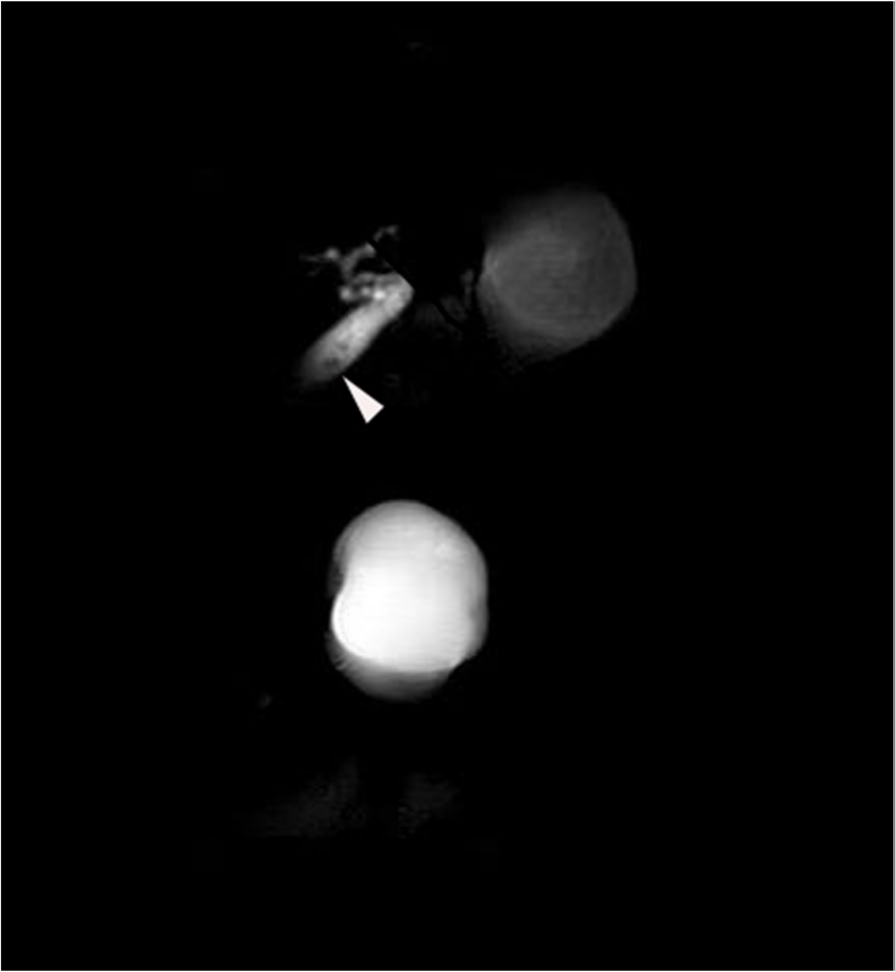
MRCP image, white arrow indicating the possible presence of common bile duct stones

After more than 2 weeks of symptomatic treatment including hepatoprotective therapy, there had been no significant improvement in the child's symptoms, signs, or liver function abnormalities. Subsequently, the girl was transferred to our hospital. The physical examination revealed the following parameters: temperature of 36.4°C, pulse rate of 123 beats per minute, respiratory rate of 30 breaths per minute, blood pressure of 92/56 mmHg, percutaneous oxygen saturation at 99%, and a weight of 5.9 kg. The infant exhibited severe jaundice without any accompanying skin rash or petechiae. Her stool appeared markedly clay-colored. No significant abnormalities were observed during the cardiac and pulmonary examinations. The abdominal examination revealed a flat abdomen with no signs of tenderness or rebound tenderness. No intra-abdominal masses were palpable. The Murphy's sign was negative. The laboratory results are presented with values as follows: bile acid (BA): 442.5 µmol/L, total bilirubin (TB): 267.8 µmol/L, conjugated bilirubin (CB): 205.7 µmol/L, alanine aminotransferase (ALT): 243 U/L, aspartate aminotransferase (AST): 436 U/L, γ-glutamyl transpeptidase (γ-GT): 1858 U/L, alkaline phosphatase (ALP): 304 U/L, blood amylase: 30 U/L, white blood cells (WBCs): 10.68×l0^9 /L, percentage of neutrophils: 15.9%, hemoglobin 87g/L and percentage of lymphocytes: 73.7%. Coagulation function test, routine urine, and stool values were within normal ranges. Furthermore, target cells were observed in the peripheral blood of this infant, and the proportion of reticulocytes was elevated (1.99%). An ultrasound examination was performed, revealing a 0.4cm diameter CBD with sedimentation. Liver fibro scan test showed significant elevation in liver stiffness (8.76kpa). Magnetic Resonance Cholangiopancreatography (MRCP) indicated suspected dilation and the presence of stones within CBD (Fig. 1).

### ERCP procedure

**Fig. 2:**
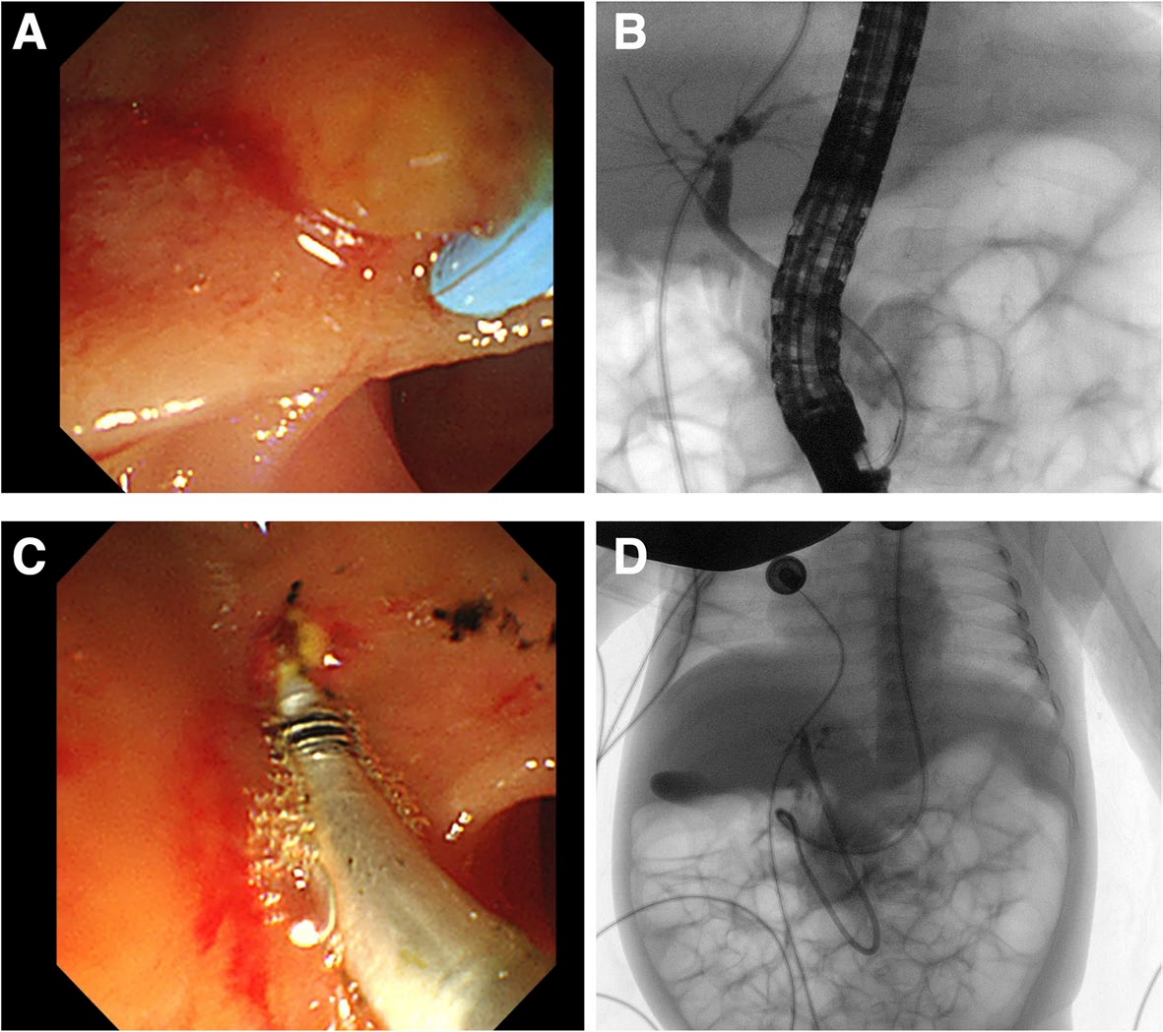
(**A**) The duodenal papilla appeared before cannulation (**B**) Under fluoroscopy, cholangiography was performed (**C**) A stone retrieval balloon was used to remove the stones in common bile duct. **D** A nasal biliary drainage catheter was placed

Considering the severe biliary obstruction in the patient, ERCP is prioritized as the initial procedure. The infant was positioned prone and underwent ERCP under general anesthesia with intubation (endotracheal tube inner diameter 3.5mm). An Olympus JF-260V duodenoscope was employed for the procedure. The duodenal papilla exhibited a papillary appearance (Fig. 2A). Successful selective biliary cannulation was achieved using a two-lumen Dreamtome™ RX Cannulating Sphincterotome (Boston Scientific) and a 0.035-inch Dreamtome™ guide wire (Boston Scientific). Cholangiography was conducted from the upper to lower segments of the CBD. The CBD had a diameter of 0.4 cm at its widest part, displaying mild dilatation without evident significant contrast filling defects (Fig. 2B). No dilatation of the intrahepatic bile duct was observed. A longitudinal incision measuring 0.2 cm was made at the 11 o’clock position on the papilla of the bile duct. Multiple sediment-like stones (Fig. 2C) were successively removed using a stone retrieval balloon (Cook Medical). Following the absence of bleeding, a 7 Fr straight-tip nasal biliary drainage catheter (Cook Medical) was inserted (Fig. 2D). The patient was transferred to the ward after the removal of the endotracheal tube, displaying stable vital signs. No post-operative complications occurred following the ERCP procedure. Laboratory results obtained one week after the ERCP procedure revealed the following values: bile acid (BA): 8.3 µmol/L, total bilirubin (TB): 66.8 µmol/L, conjugated bilirubin (CB): 56.1 µmol/L, alanine aminotransferase (ALT): 62 U/L, aspartate aminotransferase (AST): 61 U/L, γ-glutamyl transpeptidase (γ-GT): 540 U/L, alkaline phosphatase (ALP): 163 U/L, blood amylase: 48 U/L, white blood cells (WBCs): 12.41×l0^9 /L, percentage of neutrophils: 32.2%, and percentage of lymphocytes: 48.0%.

### Outcome and follow-up

Following ERCP and endoscopic nasobiliary drainage (ENBD), the girl's jaundice exhibited a marked improvement. She displayed increased energy levels and maintained a satisfactory food intake. One week post-ERCP, the nasal biliary catheter was removed, and liver function and stiffness (6.06kpa) had normalized within two weeks post-ERCP. Then the patient was discharged. Additionally, the child's hemoglobin level upon admission was 87g/L, with a elevated reticulocyte count and peripheral blood smear showing some target cells, suggesting a suspected diagnosis of hemolytic anemia. We also conducted tests for metabolic abnormalities in this patient. Her glucose-6-phosphate Dehydrogenase (G-6-PD) activity, blood tandem mass spectrometry, and urine gas chromatography showed no significant abnormalities. We also conducted whole-exome sequencing for the child and parents. The child's parents had no history of anemia or jaundice. The child carried heterozygous mutations in *UGT1A1* and *EPB41*. There were two heterozygous mutation sites in *UGT1A1*, chr2:234669144 and chr2:234665659. One mutation came from the father, and both parents carried the other mutation. The mutation at chr2:234669144 may be related to transient familial neonatal hyperbilirubinemia, but its clinical significance remains unclear. Although *EPB41* has been reported to be associated with elliptocytosis, the clinical significance of the *EPB41* mutation site chr1:29344739 in the child remains unclear. Since the child quickly recovered after surgery, with the hemoglobin level returning to normal (recovery to 111g/L two months post-ERCP.) and no recurrence of jaundice during the six-month follow-up period at the time of this case report submission, we, after consultation with the parents, decided not to further investigate the cause of hemolysis in this patient.

## Discussion

In recent years, there has been a growing body of researches dedicated to ERCP in pediatric pancreatic-biliary diseases such as pancreaticobiliary maljunction (PBM) [7, 8], pancreas trauma [9], and pancreatitis [10]. Nevertheless, pediatric ERCP remains a challenge for endoscopists due to issues related to success rates, post-operative complications, and the required medical expertise [11]. Performing therapeutic ERCP in children, particularly newborns and infants, is consistently challenging due to limitations associated with their weight and available medical devices [12]. In this study, we successfully performed a therapeutic ERCP using the JF-260V duodenoscope on an infant weighing only 5.9 kg.

Several differences exist between pediatric and adult ERCP, including variations in indications, anesthesia methods, instrumentation, and procedural techniques. Pediatric patients undergoing ERCP invariably require general anesthesia due to their limited tolerance [12]. Centers conducting pediatric ERCP procedures must possess extensive expertise in pediatric anesthesia and perioperative management. Endoscopes with a larger outer diameter may compress the trachea and have an impact on the respiratory system under general anesthesia. Therefore, monitoring and adjusting respiratory parameters during anesthesia become critically important. Additionally, the digestive tract in children is both smaller and more vulnerable. This can potentially elevate the risk of tissue edema, bleeding, and perforation.

In the majority of ERCP centers, these procedures are carried out by gastroenterologists who primarily treat adult patients. While the procedure for pediatric ERCP closely resembles that of adult ERCP, the choice of equipment and therapeutic success rates can vary across different reports [13–17]. Variations in success and therapeutic rates may depend on the proficiency of centers in managing pediatric digestive diseases and their level of perioperative care. In our center, we have conducted nearly 500 pediatric ERCP procedures with a success rate exceeding 90%. Over 80% of the pediatric ERCPs performed at our center are therapeutic. Radan Keil and colleagues reported the largest cohort, comprising 856 ERCPs conducted on 626 pediatric patients, achieving a remarkable 96% technical success rate. Within this report, 59% of the ERCPs were therapeutic [18]. ERCP procedures in patients less than 1 year of age are primarily diagnostic, aimed at excluding biliary atresia [19]. In these cases, a PJF-160 duodenoscope is consistently selected for the ERCP procedure. Sanada et al. used a PJF-160 duodenoscope and special instruments to perform therapeutic ERCP on an infant weighing 5.8kg, achieving excellent results [20]. The PJF-160 duodenoscope is currently discontinued. Therefore, performing therapeutic ERCP on infants with low weight requires the use of adult duodenoscopes, undoubtedly increasing the procedural complexity (the comparison of parameters between PJF-160 and JF-260V is shown in Table 1). To our knowledge, this infant in this case represents one of the lightest patients to undergo successful therapeutic ERCPs using adult duodenoscope in published research. In multiple reports, conventional duodenoscopes were not recommended for patients under 1 year old or weighing less than 10 kg [16, 21]. In a previous study conducted by our team, we reported a series of ERCP procedures on 15 patients under 1 year of age using the JF-260V duodenoscope, with a therapeutic rate of 88.2%. The complication rate was 11.8% (two out of 17 procedures) [22]. All complications were successfully managed with conservative treatment. Therefore, therapeutic ERCP using a conventional duodenoscope can be safely conducted under stringent and well-defined indications.

**Table 1:** The comparison of parameters between JF-260V and PJF-160

**Parameters**	**JF-260V**	**PJF-160**
**Working Length (mm)**	1240	1240
**Channel ID (mm)**	3.7	2.0
**Distal End OD (mm)**	12.6	7.5
**Insertion Tube OD (mm)**	11.3	7.5
**Depth of field (mm)**	5-60	2-60

*ID* Inner diameter, *OD *Outer Diameter

ID, Inner diameter; OD, Outer Diameter

Pediatric ERCP presents unique technical challenges. Owing to differences in the structure of the upper gastrointestinal tract between children and adults, advancing the duodenoscope during the ERCP procedure can be challenging. Particularly when the duodenoscope's body reaches the duodenum through the pylorus, the confined, narrow space poses challenges for rotating the distal end of the duodenoscope forward. Simultaneously, due to the shorter duodenum in children, the papilla is often not centrally positioned in the endoscopist's preferred field of view, frequently appearing in the upper part of the field. Furthermore, the curvature of the duodenoscope's distal end within the intestinal cavity is quite limited due to the constrained space, rendering it nearly parallel to the common bile duct (CBD). This configuration also presents challenges during cannulation and other procedures. Given the significant disparity in the disease profile between children and adults undergoing ERCP for diagnosis and treatment, pediatric patients frequently present with conditions such as PBM or congenital pancreatic duct malformations. The deep cannulation technique commonly employed in adult ERCP should not always be the primary choice for selective cannulation in pediatric patients, particularly those with PBM. Cannulation should be guided by contrast medium to prevent repeated entry into the pancreatic duct, thereby minimizing the risk of excessive damage to the delicate papilla in pediatric patients.

Based on the available literature, the incidence of complications following ERCP in children can vary between 6% and 10%, depending on the experiences of different medical centers [17]. A meta-analysis of complications following ERCP in children yielded an average complication rate of 6% [23], a figure comparable to the complication rate observed in adult ERCP procedures. Pancreatitis stands out as one of the most frequent postoperative complications [23]. Currently, the risk factors for postoperative complications following ERCP in children remain incompletely understood [24, 25]. Factors such as the experience of endoscopists, patient weight, age, choice of anesthesia method, and repeated entries into the pancreatic duct may represent potential risk factors for postoperative complications.

In this case, since the child exhibits signs of impending complete biliary obstruction, relieving the obstruction is the priority. ERCP serves both diagnostic and therapeutic purposes and is relatively minimally invasive, making it the preferred diagnostic and therapeutic approach for such patients. Besides alleviating obstruction, ERCP can also exclude surgical issues such as PBM and biliary atresia. However, considering that ERCP still has a certain false-negative rate in congenital pancreaticobiliary malformations and is more challenging in infants and young children, we may still opt for minimally invasive or open surgery when necessary. Additionally, while performing surgery, we can conduct bile duct exploration and relieve obstruction, as well as perform liver biopsy, to diagnose certain bile stasis-related diseases pathologically.

The diagnosis and treatment of this case also have certain limitations. We did not conduct the most comprehensive examinations to determine the definite causes of the patient's bile duct stones and possible hemolytic anemia. In the patient's future follow-ups, we will closely monitor the symptoms and signs and provide necessary further examinations.

In conclusion, therapeutic ERCP in pediatric patients has proven to be both safe and effective. As experience and techniques continue to evolve, it is feasible to consider implementing therapeutic ERCP procedures in low-weight infants using conventional duodenoscope. Furthermore, additional multi-center prospective studies are imperative to establish a consensus regarding the indications and standardized operating procedures for ERCP in children, particularly in the case of infant ERCP.

## Data Availability

All data generated or analyzed during this study are included in this published article.

## Abbreviations

ERCP: Endoscopic retrograde cholangiopancreatography
CBD: Common bile duct
BA: Bile acid
TB: Total bilirubin
CB: Conjugated bilirubin
ALT: Alanine aminotransferase
AST: Aspartate aminotransferase
γ-GT: γ-glutamyl transpeptidase
ALP: Alkaline phosphatase
WBC: White blood cells
MRCP: Magnetic Resonance Cholangiopancreatography
G-6-PD: Glucose-6-phosphate Dehydrogenase
